# Environmental determinants of malaria transmission in African villages

**DOI:** 10.1186/s12936-016-1633-7

**Published:** 2016-12-01

**Authors:** Noriko Endo, Elfatih A. B. Eltahir

**Affiliations:** Ralph M. Parsons Laboratory, Department of Civil and Environmental Engineering, Massachusetts Institute of Technology, Cambridge, MA 02139 USA

**Keywords:** Malaria transmission, Hydrology, Spatial impact, Characteristic time scale

## Abstract

**Background:**

Malaria transmission is complex, involving a range of hydroclimatological, biological, and environmental processes. The high degree of non-linearity in these processes makes it difficult to predict and intervene against malaria. This study seeks both to define a minimal number of malaria transmission determinants, and to provide a theoretical basis for sustainable environmental manipulation to prevent malaria transmission.

**Methods:**

Using a field-tested mechanistic malaria model, HYDREMATS, a theoretical study was conducted under hypothetical conditions. Simulations were conducted with a range of hydroclimatological and environmental conditions: temperature (t), length of wet season (T_wet_), storm inter-arrival time (T_int_), persistence of vector breeding pools (T_on_), and distribution of houses from breeding pools and from each other (X_dist_ and Y_dist_, respectively). Based on the theoretical study, a malaria time scale, T_o_, and a predictive theory of malaria transmission were introduced. The performance of the predictive theory was compared against the observational malaria transmission data in West Africa. Population density was used to estimate the scale that describes the spatial distribution of houses.

**Results:**

The predictive theory shows a universality in malaria endemic conditions when plotted using two newly-introduced dimension-less parameters. The projected malaria transmission potential compared well with the observation data, and the apparent differences were discussed. The results illustrate the importance of spatial aspects in malaria transmission.

**Conclusions:**

The predictive theory is useful in measuring malaria transmission potential, and it can also provide guidelines on how to plan the layout of human habitats in order to prevent endemic malaria. Malaria-resistant villages can be designed by locating houses further than critical distances away from breeding pools or by removing pools within a critical distance from houses; the critical distance is described in the context of local climatology and hydrology.

**Electronic supplementary material:**

The online version of this article (doi:10.1186/s12936-016-1633-7) contains supplementary material, which is available to authorized users.

## Background

The transmission dynamics of malaria are complex. Climatological, hydrological, and biological factors interact non-linearly at various stages of the transmission cycle and shape malaria transmission dynamics. Another key element of malaria transmission dynamics is environmental factors. The large dimension and non-linearity of those factors make it difficult to predict and intervene against malaria.

Malaria can be sustainably prevented through environmental modification approaches. Climatological, hydrological, and biological factors are hardly controllable; malaria treatment interventions require continuous effort to attenuate or avoid the impact of those factors. Environmental factors, on the other hand, can be manipulated; well-designed planning of environmental conditions could work as a long-term malaria prevention approach. Facing the low momentum in the public health arena of malaria control, due to fatigue of donors, spread of drug-resistance, fragile health infrastructures etc., prevention approaches, as opposed to treatment approaches, could be more sustainable and cost-effective in fighting malaria.

Planning of house locations relative to vector breeding sites and removal of pools near houses could be effective malaria prevention techniques. The importance of the relative distributions of houses and pools has been reported both in simulation studies [1, 2] and in observational studies [3–6], spelling out the large risk of contracting malaria when houses are located near vector breeding pools. The threshold distance for high malaria transmission risk is reported to be some hundreds of meters [3, 4, 6]. This paper will demonstrate that the threshold distance to prevent malaria is not universal, but significantly influenced by climatology, hydrology, and biology of local vectors, and that those factors interact in a complex manner. Planning of malaria-resistant village, thus, may not be straightforward.

This study seeks both to reduce the dimension of malaria transmission determinants, and to aid environmental manipulation to prevent malaria. The objective of this paper is to provide a tool to analyse malaria transmission potential at various environments, including different house distribution conditions. The analyses are conduct for conditions where mosquito populations are limited primarily by water availability, such as the fringe of Sahel. Based on theoretical studies with a wide range of forcings and parameters, a predictive theory of malaria transmission potential will be presented. The theory will then be tested for West Africa. In testing the theory, the impact of house distribution is emphasized.

## Methods

### Malaria transmission simulation model

This study was conducted using a mechanistic malaria transmission model, HYDREMATS—HYDRology, Entomology, and MAlaria Transmission Simulator. HYDREMATS is an agent-based model calibrated for relatively dry regions in West Africa [7–9]. HYDREMATS features the explicit representation of the location and the persistence of vector breeding pools, and the location of human houses. The spatial and temporal settings of the simulations are explained below.

### Spatial settings of simulation domain

**Fig. 1:**
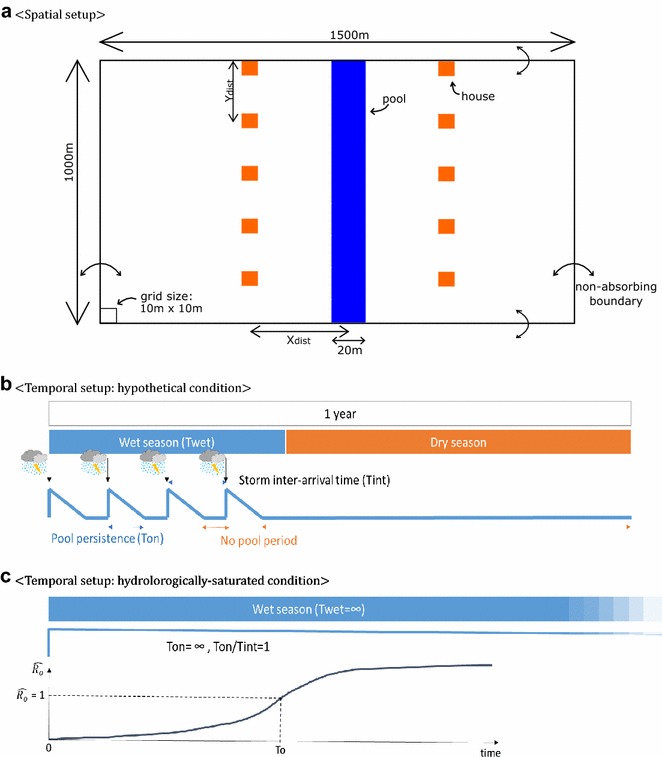
Spatial and temporal setup of the model. **a** Spatial setting in the simulations. Simulations were conducted under a 1000 m × 1500 m domain with 10 m × 10 m grids. A 20 m-width pool appears at the center of the domain. Houses are located X_dist_ away from the pool, and Y_dist_ away from each other. **b** Temporal setting: hypothetical condition. The hypothetical condition used in this study defines a clear wet and dry season in a year. During a wet season (T_wet_), pools are created at every storm inter-arrival time (T_int_), and persist for T_on_ days. Simulations were conducted for 4 years repeating the specified hypothetical hydrological conditions. **c** Temporal setting: hydrologically- saturated condition. The hydrologically saturated condition defines the condition where T_wet_ = ∞ and T_on_/T_int_ = 1. The persistent pool is assumed to be created at time zero. Under this condition, the time required to reach ${\hat{\text{R}}}_{\text{o}} = 1$ is defined as T_o_

Simulations were conducted under hypothetical settings. The spatial setup was a 1000 by 1500 m domain, having at the center a 20 m-wide breeding pool expanding longitudinally (Fig. 1a). The pool appears and disappears depending on the hydrological setting assumed, which is described in the following section. Houses are located at X_dist_ m away from the pool transversely at both sides of the pool, and Y_dist_ m away from each other longitudinally.

### Temporal settings of pools

The temporal settings employed are described in Fig. 1b. This study assumed that a year has a clear wet season for T_wet_ days and a dry season for the rest of the year. Pools appear intermittently at an interval of T_int_ days during a rainy season of T_wet_ days. T_int_ represents the storm inter-arrival time. Out of T_int_ days, pools persist for T_on_ days, gradually decreasing the probability of mosquitoes laying eggs and imposing persistence-dependent larval mortality, representing progressive disappearance of pools. Each experiment was run for the simulation period of four years, repeating the temporal setting described above.

A certain minimum number of mosquitoes were allowed to exist throughout simulations in order to seed mosquito population dynamics for the first and the subsequent rainy seasons after long dry periods. The parameter values used in the simulations are summarized in Table 1.

**Table 1:** Model parameters

Variable	Parameter values	Additional values in sensitivity study	Unit
X_dist_	50, 100, 150, 200, 250, 300	–	m
Y_dist_	50	70, 30, 10	m
T_wet_	1, 2, 3, 4, 5, 6, 8, 12	–	month
T_on_	6, 9, 12, 15, 18, 21, 27	(depend on T_int_)	day
T_int_	28	21, 14	day
Temperature (t)	19, 21, 23, 25, 27, 29, 31, 33, 35	(21, 27, 33 only)	°C

### Basic reproduction rate, R_o_

The evaluation of the malaria transmission potential under specified conditions was based on the basic reproduction rate (R_o_), which is defined as the number of infections expected to be generated by a single infectious person in a totally susceptible population. The mechanistic structure of HYDREMATS is most suitable for estimating R_o_ under dynamic conditions; conventional Ross-MacDonald formula [10] assumes static conditions. R_o_ values for a given condition were estimated in HYDREMATS to follow the exact definition of R_o_. HYDREMATS can simulate the status of each human, as well as each mosquito, and track infections (which human infects which mosquito, and which mosquito infects which human; hence it can calculate how many people were infected from an infectious person). In this way, HYDREMATS can calculate R_o_ for any dynamic environmental conditions and mosquito populations.

Long-term average of R_o_ (hereafter ${\hat{\text{R}}}_{o}$) was calculated as follows, where malaria endemics was defined as ${\hat{\text{R}}}_{o} \ge 1$.

$${\hat{\text{R}}}_{\text{o}} = \sum\limits_{i = 1}^{{N_{inf} }} {R_{o}^{i} /N_{inf} } ,$$

where N_inf_ is the number of infectious people simulated over the simulation period of four years, and ${\text{R}}_{\text{o}}^{\text{i}}$ is the number of infections generated from the i-th infectious person, assuming the population is susceptible. Because the calculation of R_o_ requires the assumption of fully susceptible population, any dual infection was counted towards ${\text{R}}_{\text{o}}^{\text{i}}$. The number of simulated malaria cases originated from the i-th infectious person, thus, differs from ${\text{R}}_{\text{o}}^{\text{i}}$. Note that N_inf_ increases with time and that ${\text{R}}_{\text{o}}^{\text{i}}$ is dictated by the time-varying vector population and environmental conditions. ${\hat{\text{R}}}_{o}$ can also be defined as the average of ${\text{R}}_{\text{o}}^{\text{i}}$ values.

### Time scale for stable malaria transmission, T_o_

This study introduces a new malaria time scale, T_o_. T_o_ (t) is defined as the length of the rainy season required to reach ${\hat{\text{R}}}_{\text{o}} = 1$ under the condition of permanent water body (hereafter hydrologically-saturated condition) at a given temperature *t* and a given spatial condition﻿ (Fig. 1c)﻿. The hydrologically-saturated condition assumes a persistent pool (i.e., T_wet_ = ∞, T_on_ = ∞ and T_on_/T_int_ = 1) that is created at the beginning of the simulation period. T_o_ is thus not a function of hydrological variables: T_wet_, T_int_, T_on_. Under this condition, ${\hat{\text{R}}}_{\text{o}} = 1$ keeps rising until the system becomes stable. The stability point may or may not exceed ${\hat{\text{R}}}_{\text{o}} = 1$. If the system stabilizes at ${\hat{\text{R}}}_{\text{o}} < 1$, T_o_ was defined as infinite. T_o_ may or may not exceed 1 year.

With t^lrv^, t^ad^, and t^para^ being *temperatures* that influence larval development time, adult life span, and parasite development time, respectively, T_o_ (t) was defined as

$${\text{T}}_{\text{o}} ({\text{t}}) = {\text{T}}_{\text{o}} ({\text{t}}^{\text{lrv}} ,{\text{t}}^{\text{ad}} ,{\text{t}}^{\text{para}} ),$$

where this study assumes t = t^lrv^ = t^ad^ = t^para^. One can also define t^lrv^ as water temperature, and t^ad^ and t^para^ as air temperature. In this study, water and air temperatures were assumed to be equal.

In order to analyse the contributions of larval development time, adult life span, and parasite development time at different temperatures, $T_{o}^{lrv} (t),T_{o}^{ad} (t),T_{o}^{para} \left( t \right)$ were introduced:

$$T_{o}^{lrv} \left( {\text{t}} \right) = {\text{T}}_{\text{o}} ({\text{t}},{\text{t}}_{\text{ref}} ,{\text{t}}_{\text{ref}} ),$$

$$T_{o}^{ad} \left( {\text{t}} \right) = {\text{T}}_{\text{o}} ({\text{t}}_{\text{ref}} ,{\text{t}},{\text{t}}_{\text{ref}} ),$$

$$T_{o}^{para} \left( {\text{t}} \right) = {\text{T}}_{\text{o}} ({\text{t}}_{\text{ref}} ,{\text{t}}_{\text{ref}} ,{\text{t}}),$$

where t_ref_ is a reference temperature set at 27 °C.

### Two dimensionless parameters

Two dimensionless parameters are introduced: D_1_ = T_wet_/T_o_ and D_2_ = T_on_/T_int_.

D_1_ measures the relative length of the wet season, T_wet_, compared to the malaria time scale, T_o_. Relatively small D_1_ indicates a wet season that is too short to create the critical size of mosquito population that may sustain malaria transmission.

D_2_ describes the variability in hydrologic conditions determining the intermittency of pools. It indicates the proportion of time that pools exist during a rainy season, and also the deviation between the condition during a rainy season and under the hydrologically-saturated condition. D_2_ nears one when pools are persistent, and zero when pools are short-lasting. As D_2_ reaches one, the condition becomes closer to the hydrologically-saturated condition (D_2_ = 1 deviates slightly from the hydrologically-saturated condition, where T_on_ = ∞, because finite Ton values impose gradual reduction in oviposition probability and the additional larval mortality).

### West Africa as a testing site

The results of the theoretical work were tested against observational data over West Africa. West Africa was selected as a testing site for the following three reasons. First, the largest burden of malaria is in Africa (e.g., 90% of deaths), of which sub-Saharan West Africa experiences disproportionately high mortality and morbidity [11]. Second, West Africa features strong North–South gradients in temperature and rainfall, resulting in a range of climate conditions and malaria vulnerability [9, 12, 13] (Additional file 1). Finally, detailed hydrological data are available for West Africa from Yamana’s study [9]. HYDREMATS has been calibrated successfully in previous studies [7–9] over the semi-arid climate in West Africa.

### Estimation of model parameters for West Africa

Hydroclimatological data and population data were used to estimate the dimensionless parameters, D_1_ and D_2_, which were then applied to estimate malaria transmission potential. Hydroclimatological data used were monthly temperature and rainfall data from 2001 to 2010 from CRU TS 3.21 [14]. Population density (*PD*) data were obtained from Gridded Population of the World, Version 3 (GPWv3) for the year 2000 [15].

The parameters used for estimating D_1_ and D_2_ are summarized in Table 2. D_1_ was obtained using T_wet_ and T_o_. T_wet_ is the length of the rainy season. In the observational data, a rainy season was defined as the period from when annual cumulative rainfall exceeds 10% until when it reaches 95%. This definition works well for West Africa because this region experiences just one distinct rainy season per year (Additional file 2). In estimating the malaria time scale, T_o_, information on temperature, X_dist_, and Y_dist_ are required. The temperature used was the average temperature over the rainy season, because this is the major period when mosquito population dynamics and malaria transmission occur.

**Table 2:** Model parameter estimation

Dimension-less number	Environmental parameters	Parameter estimation
D_1_	T_wet_	Rainy season length, during which annual cumulative rainfall is >10% and <95%
	T_o_	
	t	Rainy season average temperature
	X_dist_	Implied from population density
	Y_dist_	Set at constant
D_2_	T_on_	Estimated as T_on_/T_int_ from the empirical relationship
	T_int_	

Estimation of X_dist_ and Y_dist_ is not easy, because houses do not line up as assumed in the hypothetical setting in this study. For simplicity, Y_dist_ was set at a fixed value of 50 m. X_dist_ was estimated by comparing the observational *PD* (Additional file 3) and the *PD* implied from X_dist_ (*PD*_*imp*_(X_dist_)). The implied relationship between *PD*_*imp*_ and X_dist_ was derived assuming the following: (a) five people live in a house, (b) household density of a village is 1/X_dist_^2^ (per m^2^), and (c) a fraction (1/α) of an area is populated by villages; *PD*_*imp*_ = 5 × X_dist_^−2^ × α^−1^ × 10^−6^ (per km^2^). α was set at 15, so that the distribution of *PD* values can be captured more accurately within the X_dist_ values used in this study. Using the observational *PD*, X_dist_ was inferred (Additional file 3):

$$X_{dist} = \left\{ {\begin{array}{l} {50\quad \;\,if \,PD \ge PD_{imp} \left( {X_{dist} = 50} \right) } \\ {100\quad if \,PD \ge PD_{imp} \left( {100} \right)\;and\; PD < PD_{imp} (50) } \\ {150\quad if \,PD \ge PD_{imp} \left( {150} \right)\;and\; PD <PD_{imp} (100)} \\ {200\quad if \,PD \ge PD_{imp} \left( {200} \right)\;and\; PD <PD_{imp} (150)} \\ {250\quad if \,PD \ge PD_{imp} \left( {250} \right)\;and\; PD <PD_{imp} (200)} \\ {300\quad if \,PD \ge PD_{imp} \left( {300} \right)} \\ \end{array} } \right.$$

D_2_ is the proportion of pool persistence (T_on_) to the storm inter-arrival time (T_int_). In obtaining D_2_, instead of estimating T_on_ and T_int_ separately, the values of T_on_/T_int_ were estimated. Although pool persistence was simulated explicitly for West Africa in Yamana’s study [9], determining representative T_on_ values is challenging because natural climate forcing is neither regular nor periodic, and because T_on_ values vary significantly for each storm event. However, the total of T_on_ over the rainy season, ${\text{T}}_{\text{on}}^{\text{tot}}$, can be obtained easily without any need to differentiate storm events. The total of T_int_ over the rainy season, on the other hand, equals T_wet_ (Fig. 1b). Thus, in the hypothetical simulation setting, the proportion of ${\text{T}}_{\text{on}}^{\text{tot}}$ to T_wet_ equals the proportion of T_on_ to T_int_ (i.e., ${\text{T}}_{\text{on}}^{\text{tot}} /{\text{T}}_{\text{wet}} = {\text{T}}_{\text{on}} /{\text{T}}_{\text{int}}$). Moreover, although ${\text{T}}_{\text{on}}^{\text{tot}}$ data are available only for the twelve sites used in Yamana’s study [9], a strong correlation between ${\text{T}}_{\text{on}}^{\text{tot}} /{\text{T}}_{\text{wet}}$ and *annual*-*rainfall*/Twet was found (Additional file 4). ${\text{T}}_{\text{on}}^{\text{tot}} /{\text{T}}_{\text{wet}}$ was found to scale empirically with *annual*-*rainfall*/T_wet_, with a factor of proportionality equal to 0.14.

Thus, D_2_ can be estimated from a globally available rainfall data set. The empirical equation relating D_2_ and rainfall data reads:

$${\text{D}}_{ 2} = {\text{T}}_{\text{on}} /{\text{T}}_{\text{int}} = {\text{T}}_{\text{on}}^{\text{tot}} /{\text{T}}_{\text{wet}} = annual{\text{-}} rainfall/{\text{T}}_{\text{wet}} \times 0. 1 4$$

Although information on T_on_ and T_int_ is not usually available separately, Yamana’s study [9] provides reliable estimates for T_on_/T_int_ or D_2_.

## Results

### Timescale for stable malaria transmission

A time scale for malaria transmission, T_o_, was introduced. T_o_ was defined as the time that T_wet_ is required to last to reach ${\hat{\text{R}}}_{\text{o}} = 1$ under the hydrologically-saturated condition. In other words, if a static water body exists for T_o_ days of a year, annual R_o_ would reach the critical value of one, and malaria transmission is stable. T_o_ is thus independent of hydrological factors, T_wet_, T_on_ and T_int_, but is dependent on temperature and spatial factors, X_dist_ and Y_dist_. T_o_ was obtained for each environmental condition, i.e. for different combinations of temperature, X_dist_, and Y_dist_, by running simulations under the hydrologically-saturated condition.

**Fig. 2:**
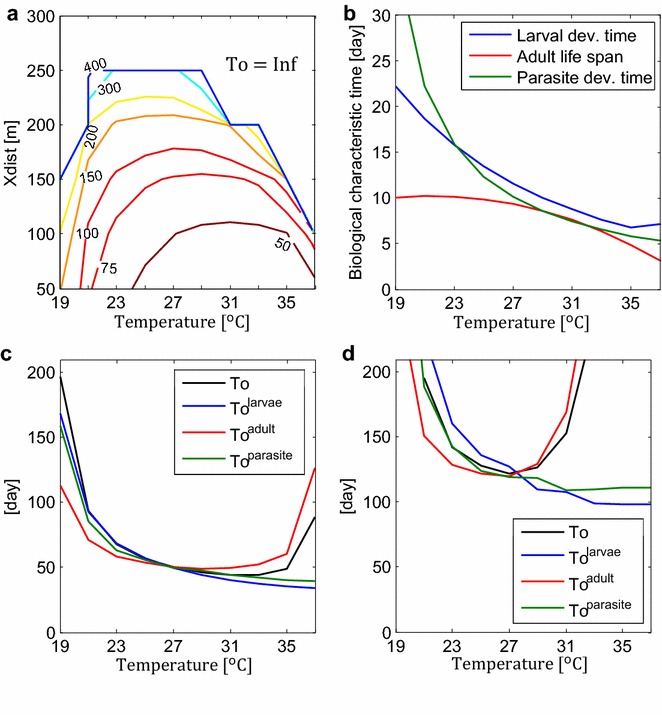
Malaria time scale and contributing biological factors. **a** Contour lines of malaria time scale, T_o_. Contour lines of T_o_ were drawn at intervals of 25 days from T_o_ = 50 to 100, of 50 days from T_o_ = 100 to 200, and of 100 days beyond that. Beyond the *blue line*, T_o_ become infinite, where the system stabilises at ${\hat{\text{R}}}_{\text{o}} < 1$. Y_dist_ was set constant at 50 m. **b** Characteristic biological time scale. Average larval development time (aquatic stage life time from eggs to adult emergence) (*blue*), adult life span (*red*), and parasite development time (EIP) (*green*) are shown as a function of temperature. **c** T_o_ and contributing biological factors at X_dist_ = 100 m. T_o_ (*black*), ${\text{T}}_{\text{o}}^{\text{lrv}}$ (*blue*), ${\text{T}}_{\text{o}}^{\text{ad}}$ (*red*), and ${\text{T}}_{\text{o}}^{\text{para}}$ (*green*) are shown. Y_dist_ was set at 50 m. **d** T_o_ and contributing biological factors at X_dist_ = 200 m. T_o_ (*black*), ${\text{T}}_{\text{o}}^{\text{lrv}}$ (*blue*), ${\text{T}}_{\text{o}}^{\text{ad}}$ (*red*), and ${\text{T}}_{\text{o}}^{\text{para}}$ (*green*) are shown. Y_dist_ was set at 50 m. T_o_ (t) = T_o_ (t^lrv^, t^ad^, t^para^), where t = t^lrv^ = t^ad^ = t^para^. ${\text{T}}_{\text{o}}^{\text{lrv}} = {\text{T}}_{\text{o}} ({\text{t}},{\text{t}}_{\text{ref}} ,{\text{t}}_{\text{ref}} )$, which is a function of only t^lrv^. ${\text{T}}_{\text{o}}^{\text{ad}} = {\text{T}}_{\text{o}} ({\text{t}}_{\text{ref}} ,{\text{t}},{\text{t}}_{\text{ref}} )$, which is a function of only t^ad^. ${\text{T}}_{\text{o}}^{\text{para}} = {\text{T}}_{\text{o}} \left( {{\text{t}}_{\text{ref}} ,{\text{t}}_{\text{ref}} ,{\text{t}}} \right)$, which is a function of only t^para^. t^lrv^, t^ad^, and t^para^ are temperatures that affects aquatic stage development, adult longevity, and parasite development, respectively. t_ref_ is a reference temperature set at 27 °C

The contour lines of T_o_ are shown in Fig. 2a on the plane of temperature and X_dist_ fixing Y_dist_ at 50 m (results for different Y_dist_ can be found in Additional file 5). Smaller T_o_ indicates higher malaria potential, which is always the case for smaller X_dist_ values. Above critical values of X_dist_, ${\hat{\text{R}}}_{\text{o}}$ never reaches one, and hence T_o_ can be assumed as infinite. The condition with T_o_ = ∞ means that malaria cannot be sustained under this environmental setup even with the existence of a permanent water body.

Responses of T_o_ to X_dist_ and temperature are non-linear, whose behaviours can be understood by looking at governing biological factors. Figure 2b illustrates the three important biological characteristic times: larval development time (blue), adult life span (red), and parasite development time in mosquito midgut (a.k.a. extrinsic incubation period, EIP) (green). Larval development time spans mosquitoes’ aquatic life from eggs, to larvae, to pupae, and to adult emergence. In this model, the larval development time follows the Depinay’s equation [16], and the adult life span follows Martens’ equation [17]. The EIP is calculated using Detinova’s equation [18]. Because these biological time scales do not translate linearly to malaria transmission potential, the behaviour of T_o_ differs from that of these timescales. In addition, the importance of these biological factors varies not just with temperature, as often asserted in the literature, but also with spatial collocation of pools and houses (Fig. 2c, d). The contributions of larval development time, adult life span, and EIP were expressed in line with T_o_, as $T_{o}^{lrv}$, $T_{o}^{ad}$, and $T_{o}^{para}$, respectively (for definition, see caption of Fig. 2 and “Methods” section). At small X_dist_, malaria transmission is limited by EIP (Fig. 2c); even though infected mosquitoes find hosts, they are not often infectious yet. This effect is stronger at colder temperatures, when EIP is large. At large X_dist_, mosquito life span is the main limiting factor for malaria transmission (Fig. 2d). Mosquitoes have to fly at least X_dist_, between houses and pools, three times within their life time to transmit malaria. This limitation is especially strong when mosquitoes’ life span is short at high temperature.

### Universality in malaria endemic conditions in dimension-less space

The conditions for ${\hat{\text{R}}}_{\text{o}} = 1$ were mapped in a dimension-less space constructed by D_1_ = T_wet_/T_o_ and D_2_ = T_o_/T_int_.

**Fig. 3:**
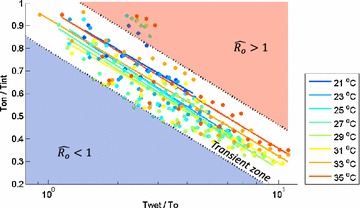
${\hat{\text{R}}}_{\text{o}} = 1$ contour lines for different temperatures. Observed points for ${\hat{\text{R}}}_{\text{o}} = 1$ (*circles*) were fitted with natural logarithmic functions (*solid lines*) on the non-dimensional D_1_–D_2_ space for each temperature, where D_1_ = T_wet_/T_o_ and D_2_ = T_on_/T_int_. The *x-axis* in this figure is shown on a logarithmic scale. Each color represents a different temperature, which applies both to the circles and the lines. No line was drawn for 19 ◦C because almost no combination of conditions lead to ${\hat{\text{R}}}_{\text{o}} > 1$. A natural logarithmic function fitted to all the observed points of ${\hat{\text{R}}}_{\text{o}} = 1$ reads as D_2_ = −0.27 log D_1_ + 0.94 (≡f_o_), and the area ±1.5 in *y-direction* from it is denoted as transient zone. Note that the figure contains the results from all the values of T_wet_, T_on_ and X_dist_ tested, as well as temperatures

Simulations were conducted with various spatial settings and hydrological conditions at different temperatures. In Fig. 3, the conditions that led to ${\hat{\text{R}}}_{\text{o}} = 1$ were presented in the dimension-less space, for all the listed values of X_dist_, T_wet_, T_on_, and temperature. The values of Y_dist_ and T_int_ were fixed in Fig. 3, but the results for different values of Y_dist_ and T_int_ can be found in Additional file 6. The observed points for ${\hat{\text{R}}}_{\text{o}} = 1$ (circles) were fitted with natural logarithmic functions (solid lines) for each temperature, which serves as contour lines for ${\hat{\text{R}}}_{\text{o}} = 1$. A logarithmic function fitted with all the presented conditions across temperature values reads D_2_ = −0.27 log D_1_ + 0.94 (≡f_o_); the area bounded by the black dotted lines in Fig. 3 shows $\pm$1.5 in y-direction from the fitted line, f_o_. The conditions above the contour line for a given temperature have high probability of having malaria endemics, where ${\hat{\text{R}}}_{\text{o}} > 1$, and the conditions that fall below the lines are likely to be malaria-free, where ${\hat{\text{R}}}_{\text{o}} < 1$.

The figure demonstrates that the conditions for ${\hat{\text{R}}}_{\text{o}} = 1$ fall in a narrow area (*transient zone*) bounded by D_2_ = f_o_ + 1.5 and D_2_ = f_o_ − 1.5 in the D_1_–D_2_ space, independent of temperature. Note that Fig. 3 aggregates the results from not only all the temperatures but also all the values of X_dist_, T_wet_, and T_on_ tested. The results for different Y_dist_ and T_int_ revealed the same characteristics (Additional file 6). On the other hand, when plotted in the T_wet_–T_on_ plane (Additional file 7), the complex and non-linear contributions of X_dist_ and temperature were apparent. With the introduction of D_1_ and D_2_, a universality of the conditions for ${\hat{\text{R}}}_{\text{o}} = 1$ was found in this theoretical study.

### Malaria transmission SCORE

The estimates of malaria transmission potential are presented as *SCORE*, based on the universal relationship found between D_1_ and D_2_. A malaria transmission score, s(D_1_, D_2_), was first calculated for a given year in the following way:

$$ s\left( {D_{1} , D_{2} } \right) = \left\{ {\begin{array}{*{20}l} {1\qquad if \; D_{2} > f_{o} \left( {D_{1} } \right) + 1.5,                                    } \\ {0\qquad if \;D_{2} \le f_{o} \left( {D_{1} } \right) + 1.5\;and \;D_{2} \ge f_{o} \left( {D_{1} } \right) - 1.5,} \\ { - 1\quad \;if \;D_{2} < f_{o} \left( {D_{1} } \right) - 1.5.                                      } \\ \end{array} } \right. $$

Subsequently, 10-year average of s(D_1_, D_2_) from 2001 to 2010 was obtained as *SCORE*.

The estimates were made both with and without *PD* information. The estimate without the information of *PD*, non-adjusted-*SCORE*, was obtained by applying a fixed value of X_dist_. The estimate adjusted for *PD*, adjusted-*SCORE*, was obtained by adopting X_dist_ values inferred from population density. Note that any values of X_dist_ ≥ 300 results in s(D_1_, D_2_) = − 1. In both estimates, Y_dist_ was fixed at 50 m.

### Predictive theory of malaria transmission

The two dimension-less variables introduced are not only intuitive, but also useful in analysing malaria transmission potential because they reduce the number of parameters involved. Based on the universality found of the conditions for ${\hat{\text{R}}}_{\text{o}} = 1$, the *predictive theory* of malaria transmission is proposed: endemic malaria when $D_{2} > f_{o} + 1.5$; critical condition to sustain malaria with potential epidemics when $D_{2} \le f_{o} + 1.5$ and $D_{2} \ge f_{o} - 1.5$, in the *transient zone*; malaria phaseout when $D_{2} < f_{o} - 1.5$.

### Testing the theory for West Africa

**Fig. 4:**
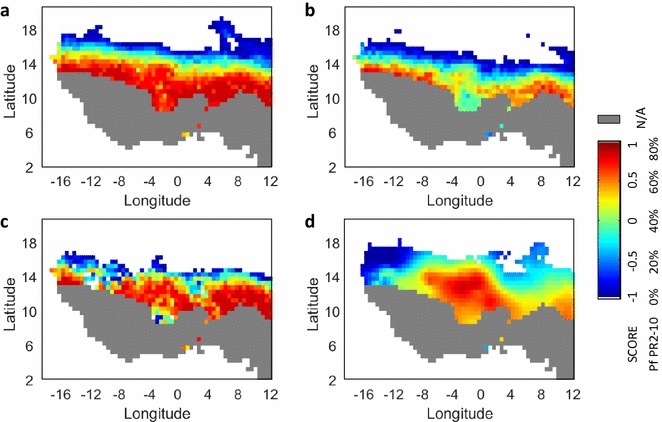
Comparisons of estimated and observed malaria trans- mission intensity. **a** Non-adjusted-SCORE at X_dist_ = 100 m. **b** Non-adjusted-SCORE at X_dist_ = 200 m. **c** Adjusted-SCORE with X_dist_ inferred from PD. The estimates of malaria transmission potential, *SCORE*, are shown in *color* with minimum and maximum values being −1 and 1. Areas with annual rainfall more than 1100 mm are not suitable for the analysis and are shown in *gray*. **d** Observed malaria intensity. *Plasmodium falciparum* parasite rate in 2- to 10-year-old children estimated for the year 2010 is shown, with minimum and maximum values being 0 and 80%. The figure was adapted from the Malaria Atlas Project (MAP) [19]. Areas with annual rainfall more than 1100 mm are also masked in *gray*

Based on the predictive theory and observed climatological data, malaria transmission potential for West Africa was estimated as *SCORE*, and compared with observational malaria transmission data (Fig. 4). *SCORE* was calculated both with and without population density information as adjusted- and non-adjusted-*SCORE*, respectively. In the adjusted-*SCORE*, population density data were used to extract spatial information; higher population density was associated with smaller X_dist_. In the non-adjusted-*SCORE*, fixed values of X_dist_ were applied.

The non-adjusted-*SCORE* for X_dist_ = 100 and 200 m are shown in Fig. 4a, b, respectively. Figure 4c presents the adjusted-*SCORE*. The estimates were compared with the observational malaria transmission intensity data in Fig. 4d, which presents the *Plasmodium falciparum* parasite rate in 2- to 10-year-old children obtained from the Malaria Atlas Project (MAP) [19]. Because HYDREMATS has been calibrated only for semi-arid parts of West Africa, no estimate was made for wet regions where annual rainfall exceeds 1100 mm (shown in gray).

The non-adjusted-*SCORE* reflects climate suitability of malaria transmission. The non-adjusted-*SCORE*s (Fig. 4a, b) captured the broad spatial limits for malaria transmission; however, they missed details of regional transmission intensity differences. The adjusted-*SCORE*, which also includes the spatial information, improved the predictability of malaria transmission potential as compared to the non-adjusted-*SCORE*, which was estimated using climate factors alone. For example, lower transmission potential is expected around an area 10°E and 2°E than the non-adjusted-*SCORE* with X_dist_ = 100 m (Fig. 4a); higher potential is expected over the area between −6°E to 0°E and 12°N to 14°N than the non-adjusted-*SCORE* with X_dist_ = 200 m (Fig. 4b). These details were captured in the adjusted-*SCORE* (Fig. 4c, d). The adjusted-*SCORE*, however, overestimated the current malaria transmission patterns west to −12°E and east to 4°E.

## Discussion

The malaria transmission potential estimate compared well with contemporary observational data, when X_dist_ (hypothetical distance between pools and human habitants) was adjusted for population density (Fig. 4c). Even without adjustment for population density (Fig. 4a), the estimate performed as well as other well-known estimates, such as those of the MARA project [12].

One of the strengths of the estimates is the detailed parametrization of hydrology. Instead of the commonly used parameter of monthly or daily rainfall amount [12, 20], three hydrological variables were employed in this study: T_wet_, T_on_, and T_int_. The same amount of rainfall, but with different temporal rainfall patterns, results in different malaria transmission potentials [21]. Having a large amount of rainfall at a time followed by subsequent dry days is different from having continuous but low intensity rainfall for many days. Pool persistence, T_on_, and storm inter-arrival time, T_int_, account for both rainfall amount and temporal patterns. The information of T_on_ is rarely available from either ground observations or satellite data sets; however, a previous study [9] made it possible to estimate T_on_/T_int_ through the association of the annual rainfall and T_wet_. In reality, pools persist over a range of period. T_on_ represents the average behaviour of pools for a given hydrological condition. Some pools disappear quicker, decreasing the total area of breeding pools. The effect of such heterogeneous pool persistence was implicitly modeled through imposing declining probability of mosquitoes laying eggs and persistence-dependent larval mortality.

The length of the rainy season, T_wet_, is also an important determinant of malaria transmission, because it takes a certain time for mosquitoes to increase their populations. The effect of the rainy season length, however, is often demonstrated only implicitly, both in statistical models and in computational models. In the MARA project, Craig et al. [12] argued that malaria transmission requires 3 and 5 months of rain at relatively warm and cold regions of Africa, respectively. The MARA project, however, did not use the rainy season length itself as a model parameter, but used the 3 or 5 months as given “time windows” for calculation. The Malaria Early Warning System (MEWS) is an improved version of MARA [22], and it outputs the number of months in a year during which climate conditions are suitable for malaria transmission. This notion is similar to T_wet_; yet the importance of T_wet_ is better understood in comparison to T_o_. The close description of hydrological conditions provides the predictive theory with an advantage over other malaria transmission models.

The consideration of the spatial effect made the estimate even more accurate. This study employed the hypothetical spatial setup, and the conceptual relationship between the house-to-pool distance and population density. The hypothetical setting is relevant to villages near water-resources reservoirs, having a long shoreline as a vector breeding pool. In reality, the distribution of houses and pools varies significantly from place to place, and hence the hypothetical setup is not valid for many locations. However, the relationship between the house-to-pool distance and population density is based on an implied relationship and should represent the characteristic relationship of regions on a large scale.

The testing of the predictive theory produced a good malaria estimate over West Africa; however, it also has noteworthy shortcomings. First of all, although the inclusion of population density data improved the quality of the estimation of malaria transmission potential, the effect of population density on malaria transmission is not straightforward. This analysis assumed that having a large population density is associated with a denser house network, and so a small distance between houses and pools, resulting in large transmission potential. A large population density, on the other hand, may be linked to economic development and to enhanced malaria controls, especially when an analysis is conducted at a village scale [23, 24]. The former effect appears to be stronger than the latter at the scale of the analysis, given that the spatial patterns of malaria transmission intensity were more closely reproduced by positively associating the malaria risk and population density. However, the latter effect, the negative association between the malaria risk and population density, could be dominating in some parts of the region.

Second, the estimate did not account for past and current levels of malaria interventions and human immunity, which may explain the overestimated malaria transmission levels west to −12°E and east to 4°E (Fig. 4c, d) in this study. In Senegal, located around −16°E to −12°E, malaria parasite rate dropped significantly (by around 30%) between 2000 and 2015 due to extensive malaria control programmes [25, 26]. Around 4°E to 12°E and 12°N to 14°N is Southern Niger, where Hausa-Fulani people live. The overestimation around this region may be accounted for by the unique low-susceptibility to malaria of the Hausa-Fulani people [27]. In this sense, this estimate addressed the climatic and environmental suitability for malaria transmission in the absence of human controls. The estimate of actual malaria transmission intensity is useful; however, the climatic and environmental suitability for malaria transmission is also vital information for malaria control programmes.

T_o_ is a biological time scale introduced in this study, which is associated with the time needed to reach ${\hat{\text{R}}}_{\text{o}} = 1$ under the assumption of the hydrologically-saturated condition. The calculation of T_o_ depends on temperature and spatial settings of houses and pools but is independent of hydrological conditions, i.e., T_wet_, T_on_, and T_int_.

Malaria transmission is complex, requiring many factors to be considered. The introduction of T_o_, together with the two dimension-less variables, D_1_ = T_wet_/T_o_ and D_2_ = T_on_/T_int_ disclosed a universality in the conditions for stable malaria transmission. D_1_ and D_2_ not only reduce the number of malaria transmission determinants, but are also intuitive. Readers should, however, be aware that the values of T_o_ were obtained for relatively dry regions in West Africa. For other regions with more or less competent vectors, or with other limiting factors in play, the values of T_o_ should be evaluated accordingly.

The application of T_o_ and the predictive theory is not limited to the estimation of malaria transmission potential; it also offers guidelines regarding the minimum distances that houses and pools should be apart to prevent malaria, as shown in the following examples. Firstly, T_o_ helps in defining the house-to-pool distances needed to prevent malaria transmissions around permanent water bodies, such as water-resources reservoirs. To prevent malaria near a permanent water body, where the condition compares with the hydrologically-saturated condition, T_o_ should be infinite. T_o_ can be rendered infinite by locating houses away from pools by a certain distance, whose value depends on temperature (Fig. 2). Secondly, malaria-preventive distances between houses and pools can also be inferred for other general conditions from the predictive theory and the T_o_–X_dist_ relationship [from climatological data, T_on_/T_int_ can be estimated as described in this paper’s “Methods” section. By using the T_on_/T_int_ with the predictive theory (Fig. 3), T_wet_/T_o_ associated with Ro = 1 can be found. As T_wet_ can be obtained from climatological data, T_o_ can be calculated. Finally, applying the T_o_–X_dist_ relationship (Fig. 2a) for a given temperature, an X_dist_ value leading to the Ro = 1 condition can be found]. Malaria-resistant villages would require that houses should be located further than the critical distances, or that pools should be removed within the critical distances from houses.

The critical distances to prevent malaria found in this study also provide guidance for resource allocation. When relocation of settlements or removal of pools is not feasible, conventional interventions, such as distribution of bednets, remain primary malaria control strategy. The critical distances presented in this study shows the distance from pools within which such intervention should be prioritized. This study provides a useful guidance for estimating malaria transmission potential, and for designing malaria resistant villages by controlling house-to-pool distances. R_o_ values and critical distances suggested in this study should not be taken as absolute values that fit every condition, but they should be adjusted depending on the details of the local environment. In particular, malaria transmission potential and critical malaria-preventive distances depend on the distribution of houses and the initial number of mosquitoes in the beginning of the rainy seasons. The impact of different house distribution could be a future research topic. The sensitivity to the initial mosquito population is not the focus of this study; how and how many mosquitoes emerge after a long dry period are the questions yet to be clarified. At the limit of no mosquitoes surviving after a long dry season, the potential for malaria transmission would not exist even under the wettest hydrologic condition. The appearance of mosquito population after a long dry period can be explained either by aestivation or migration [28], and imposing the minimum mosquito population in HYDREMARS conceptually agree with the aestivation hypothesis.

## Conclusion

In fighting malaria, the use of medicine and bed-nets are effective approaches that can be deployed in malaria-endemic regions. Here, this study emphasizes alternative preventive measures and suggested that effective environmental planning may offer a more sustainable approach that addresses the root cause of the problem. In order to reduce the dimension of malaria transmission determinants, and to aid environmental manipulation for malaria prevention, the predictive theory was developed and tested against observations.

The predictive theory, constructed with the two dimensionless parameters, is useful in measuring malaria transmission potential. The two dimensionless parameters contain information of many hydroclimatological and environmental factors. The comparison between the malaria estimate from the predictive theory and the observational data illustrated the importance of the spatial collocation of vector breeding pools and human habitats.

The predictive theory can provide guidelines on how to plan the layout of human habitats in order to prevent endemic malaria. Malaria-resistant villages can be designed by locating houses further than critical distances away from breeding pools or by removing pools within a critical distance from houses; the critical distance is described in the context of local climatology and hydrology.

## Additional files

**Additional file 1.** Malaria intensity and hydroclimatological conditions in West Africa.

**Additional file 2.** Cumulative rainfall and T_wet_ for twelve sites in West Africa.

**Additional file 3.** Population density and inferred X_dist_ in West Africa.

**Additional file 4.** Comparison between Tontot /T_wet_ and *annual-rainfall*/T_wet_.

**Additional file 5.** Contour lines of T_o_ at different Y_dist_ values.

**Additional file 6.** Conditions for ${\hat{\text{R}}}_{\text{o}} = 1$ and the sensitivity to X_dist_, Y_dist_ and T_int_.

**Additional file 7.** ${\hat{\text{R}}}_{\text{o}} = 1$ contour lines on the plane of T_on_ and T_wet_.

## Abbreviations

HYDREMATS: hydrology, entomology, and malaria transmission simulator
R_o_: basic reproduction rate
$\hat{R}_{o}$: long-term average of R_o_ calculated in HYDREMATS. $\hat{R}_{o} = \left( {\sum\nolimits_{i = 1}^{{N_{inf} }} {R_{o}^{i} } } \right)/N_{inf}$
$R_{o}^{i}$: the number of infections generated from the i-th infectious person, assuming that the population is fully susceptible
N_inf_: the number of infectious people simulated over the simulation period of four years
X_dist_: distance between vector breeding pools and human habitats (horizontal distance) (m)
Y_dist_: distance between human habitats (longitudinal distance) (m)
T_wet_: length of the rainy season (days)
T_int_: storm inter-arrival time (days)
T_on_: pool persistence (days)
T_o_: malaria time scale. The length of the rainy season required to reach $\hat{R}_{o} = 1$ under the hydrologically-saturated condition at a given temperature and a given spatial condition. T_o_(t) = T_o_(t^lrv^, t^ad^, t^para)^, where this study assumes t = t^lrv^ = t^ad^ = t^para^ (days)
t: temperature (°C)
t^lrv^: temperature that influences aquatic-stage development (°C)
t^ad^: temperature that influences adult life span (°C)
t^para^: temperature that influences parasite development (°C)
t_ref_: reference temperature set at 27 (°C)
$$T_{o}^{lrv} \left( t \right)$$: $$T_{o}^{lrv} \left( t \right) = T_{o} (t, t_{ref} , t_{ref} )$$
$$T_{o}^{ad} \left( t \right)$$: $$T_{o}^{ad} \left( t \right) = T_{o} (t_{ref} ,t, t_{ref} )$$
$$T_{o}^{para} \left( t \right)$$: $$T_{o}^{para} \left( t \right) = T_{o} (t_{ref} , t_{ref} , t)$$
D_1_: dimensionless parameter. D_1_ = T_wet_/T_o_
D_2_: dimensionless parameter. D_2_ = T_on_/T_int_
*PD*: population density (per km^2^)
*PD*_*imp*_: implied population density (per km^2^). *PD*_*imp*_ = 5 × X_dist_^−2^ × α^−1^ × 10^−6^, with α being set at 15
s(D_1_, D_2_): malaria transmission score calculated for a year
*SCORE*: malaria transmission estimate. Ten-year average of s(D_1_, D_2_) from 2001 to 2010
EIP: extrinsic incubation period
