# Supplementary material for: Environmental determinants of malaria transmission in African villages
Source: Malar J. 2016 Dec 1;15:578. doi: 10.1186/s12936-016-1633-7 (PMC5131557; doi:10.1186/s12936-016-1633-7)
Supplement: Supplementary file 1 — Additional file 1. Malaria intensity and hydroclimatological conditions in West Africa. [file 12936_2016_1633_MOESM1_ESM.pdf]

Additional file 1. Figure S1.

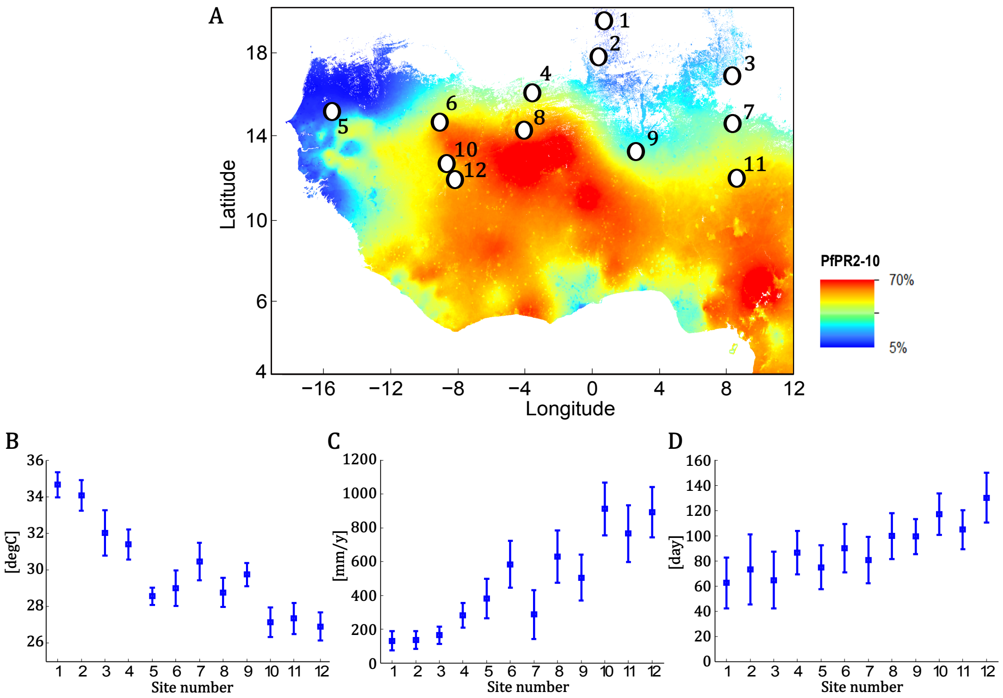

Fig. S1: Malaria intensity and hydroclimatological conditions in West Africa. (A) Malaria intensity and twelve study sites in West Africa. *Plasmodium falciparum* parasite rate in two- to ten-year-old children estimated for the year 2010 is shown (color). The figure was adapted from the Malaria Atlas Project (MAP). Also shown are the locations of twelve study sites used in Yamana [1] (circles with site number). The sites were numbered from north to south. All the twelve sites are located in semi-arid regions, where annual rainfall is less than 1100mm. **(B) Average rainy season temperature for the twelve sites. (C) Average annual rainfall for the twelve sites. (D) Average rainy season length for the twelve sites.**

[1] Yamana TK. Mechanistic modelling of the links between environment, mosquitoes and malaria transmission in the current and future climates of West Africa. PhD thesis, Massachusetts Institute of Technology, February 2015.
