# Supplementary material for: Environmental determinants of malaria transmission in African villages
Source: Malar J. 2016 Dec 1;15:578. doi: 10.1186/s12936-016-1633-7 (PMC5131557; doi:10.1186/s12936-016-1633-7)
Supplement: Supplementary file 2 — Additional file 2. Cumulative rainfall and Twet for twelve sites in West Africa. [file 12936_2016_1633_MOESM2_ESM.pdf]

Additional file 2. Figure S2.

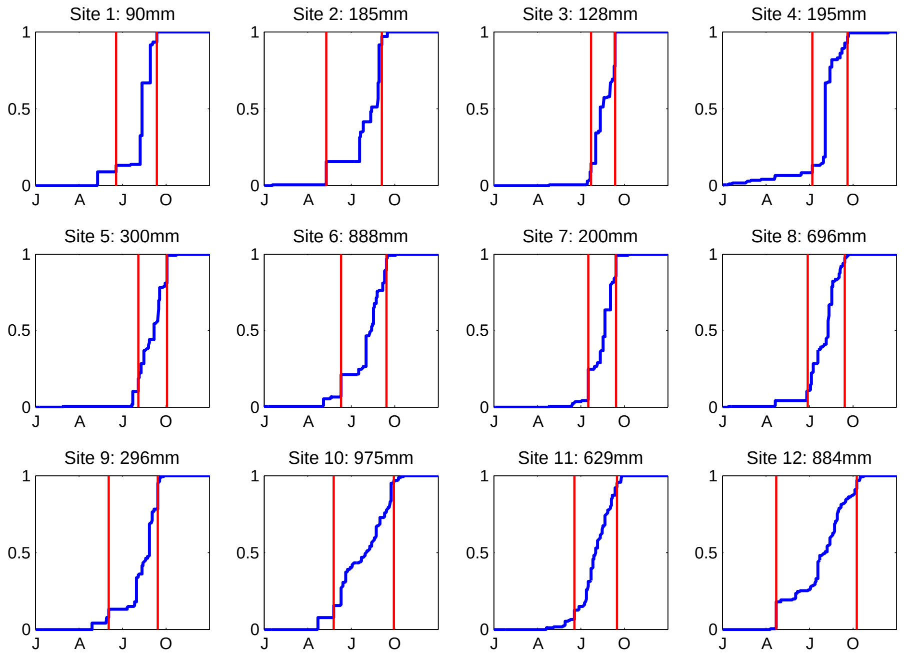

Fig. S2: Cumulative rainfall and T_{wet} for twelve sites in West Africa. Cumulative rainfall distribution for each of the twelve study sites is shown, with the x-axis being time from the beginning to the end of the year 1998. The number next to the site number indicates the annual rainfall for the year. The two red lines indicate the times when cumulative rainfall exceeds 10% and 95%, respectively, of annual rainfall. T_{wet} is the length between the two red lines. Rainfall data were obtained from CMORPH version 1.0 [1]. Note that the CMORPH data shown here are 30-minute data, but CRU data used in the analysis are monthly data.

[1] Robert J, Joyce JE, Janowiak PAA, Pingping X. CMORPH: A Method that Produces Global Precipitation Estimates from Passive Microwave and Infrared Data at High Spatial and Temporal Resolution. Journal of Hydrometeorology. 2004;5:487503.
