# Supplementary material for: Environmental determinants of malaria transmission in African villages
Source: Malar J. 2016 Dec 1;15:578. doi: 10.1186/s12936-016-1633-7 (PMC5131557; doi:10.1186/s12936-016-1633-7)
Supplement: Supplementary file 3 — Additional file 3. Population density and inferred Xdist in West Africa. [file 12936_2016_1633_MOESM3_ESM.pdf]

Additional file 3. Figure S3.

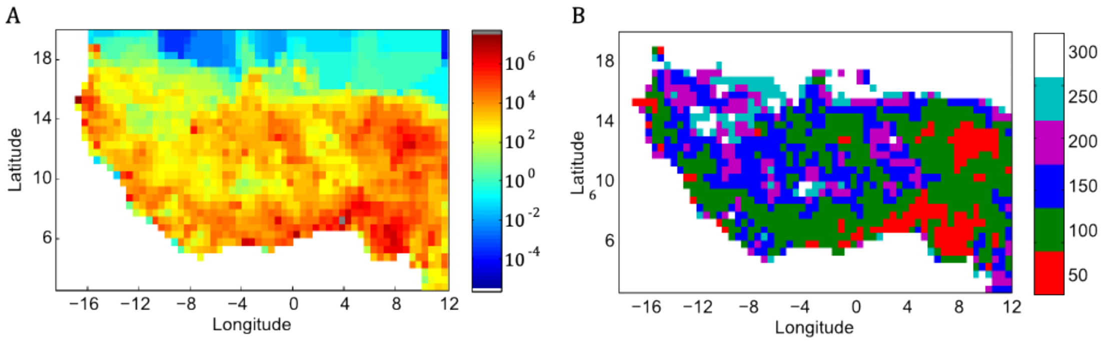

Fig. S3: Population density and inferred X_{dist} in West Africa. (A) Population density in West Africa. Population density [$per\ km^2$] is plotted in logarithmic scale. The data were obtained from Gridded Population of the World, Version 3 (GPWv3) for the year 2000 [1]. **(B) X_{dist} inferred from population density.**

[1] Columbia University. Gridded Population of the World Version 3 (GPWv3), 2005.
