# Supplementary material for: Environmental determinants of malaria transmission in African villages
Source: Malar J. 2016 Dec 1;15:578. doi: 10.1186/s12936-016-1633-7 (PMC5131557; doi:10.1186/s12936-016-1633-7)
Supplement: Supplementary file 4 — Additional file 4. Comparison between Tontot /Twet and annual-rainfall/Twet. [file 12936_2016_1633_MOESM4_ESM.pdf]

Additional file 4. Figure S4.

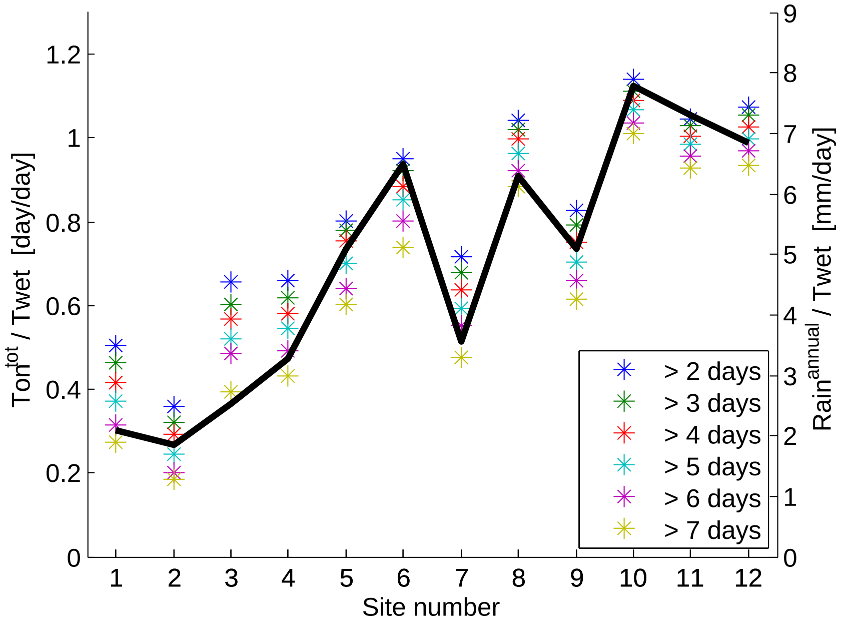

Fig. S4: Comparison between $T_{on}{}^{tot}/T_{wet}$ **and** $annual\text{-}rainfall/T_{wet}$**.** $T_{on}{}^{tot}$ was obtained from HYDREMATS simulation results by Yamana [1], by aggregating the time that the major pool in the simulation domain existed. A value was included in $T_{on}{}^{tot}$ only if the pool persisted for more than a certain number of days because the most short-lived temporary pools do not contribute to mosquito population dynamics. $T_{on}{}^{tot}$ calculation hence depends on the imposed requirement for the pool persistence. The minimum persistence of pools was set at 2 to 7 days, as indicated in the legend. The value of $T_{on}{}^{tot}/T_{wet}$ was calculated for each minimum persistence (stars), and it is shown on the left y-axis. $Annual\text{-}rainfall/T_{wet}$ is shown in black line on the right y-axis. All of $T_{on}{}^{tot}$, T_{wet}, annual-rainfall, and T_{wet} shown here are the fifteen-year average from 1998 to 2012. A significant correlation between $T_{on}{}^{tot}/T_{wet}$ and $annual\text{-}rainfall/T_{wet}$ was found.

[1] Yamana TK. Mechanistic modelling of the links between environment, mosquitoes and malaria transmission in the current and future climates of West Africa. PhD thesis, Massachusetts Institute of Technology, February 2015.
