# Supplementary material for: Environmental determinants of malaria transmission in African villages
Source: Malar J. 2016 Dec 1;15:578. doi: 10.1186/s12936-016-1633-7 (PMC5131557; doi:10.1186/s12936-016-1633-7)
Supplement: Supplementary file 5 — Additional file 5. Contour lines of To at different Ydist values. [file 12936_2016_1633_MOESM5_ESM.pdf]

Additional file 5. Figure S5.

T_o for different Y_{dist}

The introduced biological timescale, T_o, is a function of temperature and spatial distribution of pools and houses. Dependence of T_o on temperature, X_{dist}, and Y_{dist} are shown in Fig. S5. Smaller X_{dist} and Y_{dist} lead higher malaria transmission potential, shortening T_o.

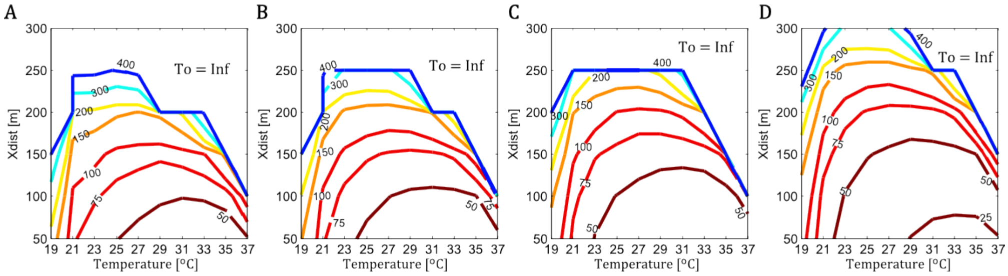

Fig. S5: Contour lines of T_o at different Y_{dist} values. Contour lines of T_o at Y_{dist} = 70m (A), 50m (B), 30m (C), and 10m (D). The contour lines of T_o were drawn at intervals of 25 days from T_o=50 to 100, of 50 days from T_o=100 to 200, and of 100 days beyond that. Beyond the blue line, T_o became infinite, where the system equilibrated at $\hat{R}_o < 1$.
