# Supplementary material for: Environmental determinants of malaria transmission in African villages
Source: Malar J. 2016 Dec 1;15:578. doi: 10.1186/s12936-016-1633-7 (PMC5131557; doi:10.1186/s12936-016-1633-7)
Supplement: Supplementary file 6 — Additional file 6. Conditions for ${\hat{\text{R}}}_{\text{o}} = 1$R^o=1 and the sensitivity to Xdist, Ydist and Tint. [file 12936_2016_1633_MOESM6_ESM.pdf]

Additional file 6. $\hat{R}_o = 1$ conditions for different Y_{dist} and T_{int}. Figure S6.

$\hat{R}_o = 1$ conditions for different Y_{dist} and T_{int}

In this section, the sensitivity of $\hat{R}_o$, especially the $\hat{R}_o = 1$ contour lines, to Y_{dist} and T_{int} is presented. The longitudinal household distance, Y_{dist}, was varied from 70m, 50m, 30m, to 10m. Sensitivity to the storm inter-arrival time, T_{int}, was tested for $T_{int} = 28$, 21, and 14 days. The results of the sensitivity tests were reported only at temperature of 27 °C; those at other temperatures revealed a similar trend.

Sensitivity to Y_{dist}

A smaller Y_{dist} means a denser household network, where mosquitoes would find humans for bloodmeals more easily. The results of $\hat{R}_o = 1$ contour lines for different Y_{dist} are shown in Fig. S6. Fig. S6Aa presents the results in the $T_{wet} - T_{on}$ space: $Y_{dist} = 70$m (dashed line), 50m (solid line), 30m (dotted line), and 10m (dash-dot line). The results with each X_{dist} value were differentiated by the color: $X_{dist} = 50$m (red), 100m (green), 150m (blue), and 200m (purple). As expected, smaller Y_{dist} values required smaller T_{on} and T_{int} to have the condition for malaria endemics, $\hat{R}_o = 1$. Yet the sensitivity to Y_{dist} varied among different X_{dist} values. Smaller X_{dist} of 50m and 100m resulted in a lower sensitivity to Y_{dist}, whereas larger X_{dist} of 150m and 200m resulted in a higher sensitivity.

When plotted in the $D_1 - D_2$ space, a universality of the conditions for $\hat{R}_o = 1$ was found (Fig. S6Ab). Y_{dist} values affect D_1 through T_o, as well as $\hat{R}_o$, but not D_2. Fig. S6Ab shows the conditions that generated $\hat{R}_o = 1$, differentiating Y_{dist} by markers (upward-pointing triangle for $Y_{dist} = 70$m, filled circle for $Y_{dist} = 50$m, square for $Y_{dist} = 30$m, and downward-pointing triangle for $Y_{dist} = 10$m), as well as X_{dist} by colors (red for $X_{dist} = 50$m, green for $X_{dist} = 100$m, blue for $X_{dist} = 150$m, and purple for $X_{dist} = 200$m). Although a certain universality in the conditions for $\hat{R}_o = 1$ can be found, stratification by X_{dist} is present. Larger X_{dist} required slightly higher values of D_1 and/or D_2 for malaria to be endemic, shifting the conditions for $\hat{R}_o = 1$ upward in the figure. Sensitivity to Y_{dist}, on the other hand, seems to be negligible when compared at the same X_{dist}. Because smaller X_{dist} and Y_{dist} lead larger malaria transmission potential, the condition for $\hat{R}_o = 1$ requires shorter T_{wet} for a given set of T_{on} and T_{int}, or D_1. However, with smaller X_{dist} and Y_{dist}, the corresponding T_o also becomes smaller, which works to counteract the requirement of shorter T_{wet} and maintains T_{wet}/T_o or $D1$. The relationship between D_1 and D_2 is thus hold.

Sensitivity to T_{int}

One of the bold assumptions in this study concerns T_{int}. Fig. S6Ba demonstrates how the $\hat{R}_o = 1$ contour lines respond to different T_{int} in the $T_{wet} - T_{on}$ space: $T_{int} =$

28 days in solid lines, $T_{int} = 21$ days in dash lines, and $T_{int} = 14$ days in dotted lines. The results with each X_{dist} value were differentiated by the color: $X_{dist} =$ 50m (red), 100m (green), 150m (blue), and 200m (purple). At our simulation setting, $T_{int} = T_{on} + T_{off}$; consequently, a smaller T_{int} for a given T_{on} means a shorter T_{off}, making malaria transmission more likely. As a result, smaller T_{int} resulted in $\hat{R}_o = 1$ contour lines that require smaller values of T_{on} and T_{wet} in general. Note that $T_{on} \leq T_{int}$, and so T_{on} could increase only up to T_{int}. In the $T_{wet} - T_{on}$ space, sensitivity to T_{int} was found significant.

Sensitivity to T_{int} was then investigated in the dimension-less space of D_1 and D_2 (Fig. S6Bb). T_{int} affects D_2 $(= T_{on}/T_{int})$, as well as $\hat{R}_o$, but not D_1. Different markers indicate different conditions of T_{int}: filled circle for $T_{int} = 28$ days as in the baseline study, diamond for $T_{int} = 21$ days, square for $T_{int} = 14$ days. Different colors indicate different X_{dist} values. Although $\hat{R}_o$ is sensitive to T_{int}, the conditions for $\hat{R}_o = 1$ on the dimension-less space line up in a good agreement among different T_{int} values, especially when compared at the same X_{dist}. A certain magnitude of deviation was found for $T_{int} = 14$ days; however, most of the conditions for $\hat{R}_o = 1$ observed fell in the *transient zone*.

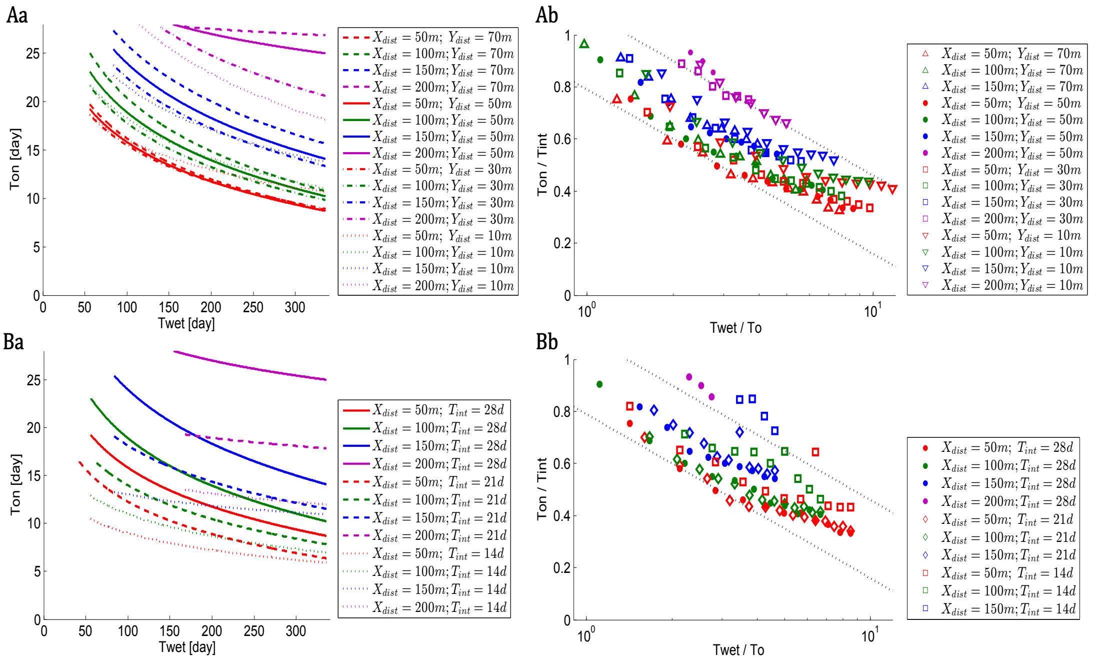

Fig. S6: Conditions for $\hat{R}_o = 1$ and the sensitivity to X_{dist}, Y_{dist} and T_{int}. (A) Conditions for $\hat{R}_o = 1$ under different Y_{dist} at 27 °C. (B) Conditions for $\hat{R}_o = 1$ under different T_{int} at 27 °C. Colors indicate different X_{dist}: red for X_{dist} =50m, green for X_{dist} =100m, blue for X_{dist} =150m, and purple for X_{dist} =200m. (Aa) Sensitivity to Y_{dist} on the T_{wet}–T_{on} space: dashed line for $Y_{dist} = 70$m, solid line for $Y_{dist} = 50$m (base value), dash-dot line for $Y_{dist} = 30$m, and dotted line for $Y_{dist} = 10$m. (Ab) Sensitivity to Y_{dist} on the D_1–D_2 space: upward-pointing triangle for $Y_{dist} = 70$m, filled-in circle for $Y_{dist} = 50$m, square for $Y_{dist} = 30$m, and downward-pointing triangle for $Y_{dist} = 10$m. The area within the dotted lines indicates the *transient zone*. (Ba) Sensitivity to T_{int} on the T_{wet}–T_{on} space: solid line for $T_{int} = 28$ days, dashed line for $T_{int} = 21$ days, and dotted line for $T_{int} = 14$ days. (Bb) Sensitivity to T_{int} on the D_1–D_2 space: filled-in circle for $T_{int} = 28$ days, diamond for $T_{int} = 21$ days, and square for $T_{int} = 14$ days. The area within the dotted lines indicates the *transient zone*.
