# Supplementary material for: Environmental determinants of malaria transmission in African villages
Source: Malar J. 2016 Dec 1;15:578. doi: 10.1186/s12936-016-1633-7 (PMC5131557; doi:10.1186/s12936-016-1633-7)
Supplement: Supplementary file 7 — Additional file 7. ${\hat{\text{R}}}_{\text{o}} = 1$R^o=1 contour lines on the plane of Ton and Twet. [file 12936_2016_1633_MOESM7_ESM.pdf]

Additional file 7. $\hat{R}_o = 1$ conditions in $T_{wet} - T_{on}$ space. Figure S7.

$\hat{R}_o = 1$ conditions in $T_{wet} - T_{on}$ space

Conditions for $\hat{R}_o = 1$ — the critical value for malaria endemics — at various T_{wet}, T_{on}, X_{dist}, and temperature were shown in the dimension-less space of D_1 and D_2 in Fig. 3, revealing a universality. The same conditions for $\hat{R}_o = 1$ were plotted on the plane of T_{wet} and T_{on} as contour lines in Fig. S7. Fig. S7A shows the contour lines of $\hat{R}_o = 1$ for different X_{dist} values at a fixed temperature of 27 °C. Fig. S7B shows the contour lines of $\hat{R}_o = 1$ for different temperatures at $X_{dist} = 100$ m. The figures illustrate that the conditions for stable malaria transmission depends on T_{wet}, T_{on}, X_{dist}, and temperature, and that the interplay of these variables is complex. The large dimension and non-linearity of malaria transmission determinants highlight the utility of the *predictive theory*.

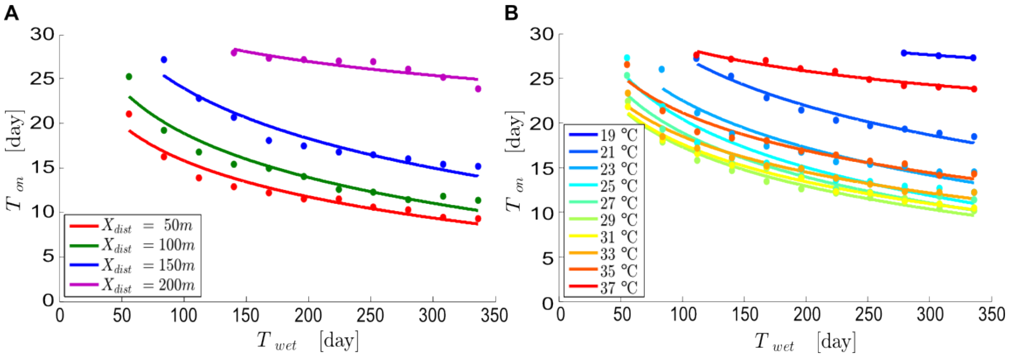

Fig. S7: $\hat{R}_o = 1$ contour lines on the plane of T_{on} and T_{wet}. (A) $\hat{R}_o = 1$ contour lines for different X_{dist} on the plane of T_{on} and T_{wet} at 27 °C. Observed points for $\hat{R}_o = 1$ (circles) were fitted with natural logarithmic functions (solid lines) on the T_{wet}–T_{on} space for each X_{dist}. **(B) $\hat{R}_o = 1$ contour lines for different temperature on the plane of T_{on} and T_{wet} at $X_{dist} = 100$ m.** Observed points for $\hat{R}_o = 1$ (circles) were fitted with natural logarithmic functions (solid lines) on the T_{wet}–T_{on} space for each temperature.
